# Characterization and evaluation of antibacterial and antiproliferative activities of crude protein extracts isolated from the seed of *Ricinus communis* in Bangladesh

**DOI:** 10.1186/s12906-016-1185-y

**Published:** 2016-07-12

**Authors:** M. Abdulla Al-Mamun, Zerin Akter, Md Josim Uddin, K. M. K. B. Ferdaus, K. M. F. Hoque, Z. Ferdousi, M. Abu Reza

**Affiliations:** Department of Genetic Engineering and Biotechnology, Protein Science Lab, University of Rajshahi, Rajshahi, 6205 Bangladesh; Department of Pharmacy, Faculty of Science and Engineering, International Islamic University Chittagong, Chittagong, 4203 Bangladesh

**Keywords:** Apoptosis, Castor bean, Disc diffusion, EAC cells, Lectin, MIC, Mice, Protein

## Abstract

**Background:**

*Ricinus communis* (*Euphorbiaceae*) has previously been reported to possess analgesic, antihistamine, antioxidant and anti-inflammatory activities. This study was designed for isolation, characterization and evaluation of antibacterial and anti-proliferative activities of *R. communis* seed protein.

**Methods:**

The concentration and molecular weight of *R. communis* seed protein were estimated by SDS-PAGE and spectrophotometric analysis, respectively. Lectin activity was evaluated by hemagglutination assay on mice blood. In vitro susceptibility of four human pathogenic bacteria including *Escherichia coli, Pseudomonas aeruginosa*, *Enterobacter aerogenes* and *Staphylococcus aureus* was detected using disk diffusion assay, and minimum inhibitory concentration (MIC) value was determined using micro-dilution method. A total of twenty four Swiss albino mice containing Ehrlich’s ascites carcinoma (EAC) cells were treated with the crude protein of *R. communis* at 50 and 100 μg/ml/d/mouse for 6 days. Growth inhibitory activity of *R. communis* seed protein on EAC cells was determined by haemocytometer counting using trypan blue dye and DAPI (4΄,6-diamidino-2-phenylindole) staining was used to assess apoptotic cells.

**Results:**

The protein concentration of six *R. communis* (castor) varieties ranged between 21–35 mg/ml and molecular weight between 14–200 kDa. Castor protein agglutinated mice blood at 3.125 μg/wall. The seed protein shows considerable antimicrobial activity against *E. coli, P. aeruginosa* and *S. aureus*, exhibiting MIC values of 250, 125 and 62.5 μg/ml, respectively. Administration of seed protein led to 54 % growth inhibition of EAC cells at 100 μg/ml. DAPI staining indicates marked features of apoptosis including condensation of cytoplasm, nuclear fragmentation and aggregation of apoptotic bodies etc.

**Conclusion:**

Our study suggests that the lectin rich *R. communis* seed protein has strong antibacterial and anticancer activities.

## Background

Pathogenic bacteria have always been considered as a major cause of death worldwide especially in developing countries [1]. Though over the past century, antimicrobial drugs effectively neutralized the pathogenic attack but their therapeutic potentiality are now under great risk as many frequently used antibiotics have already became less effective against certain superbugs. The recurrent ineffectiveness of the conventional therapeutic agents has occurred not only due to the secretion of novel microbial toxins but also for the emergence of multidrug resistant strain of bacteria, showing negligible susceptibility to current antibiotics [2]. Moreover, random application of conventional antibiotics induces the micro-organisms to be mutated as well as impart several unwanted side effects on public health such as hypersensitivity, allergic reaction, immune suppression etc [3].

Cancer is the most common deadly disease in the current world with high rate of mortality and morbidity. In 2012, an estimated 14.1 million new cases of cancer occurred and 8.2 million deaths worldwide [4]. The difficulty to get the fruitful outcome of cancer treatment is mainly due to some defects in cellular signals involved in the regulation of cell cycle check point which lead the cells to proliferate abnormally [5]. More precisely, the cells gain the capacity to evade from program cell death (apoptosis). Furthermore, cancer patients are more prone to bacterial infection that one study shows that about 38 % of lung cancer patients with febrile neutropenia are identified as infected with bacteria mainly Gram negative category [6]. It is estimated that over 15 % of malignancies worldwide can be attributed to infections [7]. Bacterial infection often involves at incipient stage of cancer formation. The toxins resultant from chronic infection disturbs the cell cycle resulting in altered cell growth or resistance to apoptosis which lead to tumorigenesis [8]. Sometimes bacterial infection leads to progression of cancer by evading host immune system which contributes to carcinogenic changes through the stimulatory and mutagenic effects of cytokines released by inflammatory cells [9]. Immunosuppression resulting from current cancer treatment such as chemotherapy and radiotherapy leads the cancer patient to prone to bacterial infection [10]. Therefore, the development of a common treatment, having both antibacterial and anticancer potential would reduce the treatment related expenditure as well as save numerous lives.

Plants are the rich source of different phytochemicals with various bio-structures and potent bioactivities against a number of diseases including cancer and bacterial diseases. More than 80 % of the total world’s population relies on herbal medicine to meet their primary health care needs [11]. Current pharmaceutical industries are depend to a large extant on natural compound as a rich source of potential drug candidates and statistics show that over 60 % of the current anticancer drugs are related with herbal product as their origin [12]. Plant based bioactive compounds generally neutralize the bacterial attack as well as exerts their anti-proliferative action only to the target cells, rather than affecting to the host cells [13, 14], which increases the demand of phytochemicals as the potential lead compounds. Therefore, exploring novel plant derived medicinal agents would contribute to manage the growing problem of drug resistance and toxicity of commercially available antibiotics.

*R. communis* is a soft wooden flowering perennial shrub, commonly known as castor, widely distributed in tropical and subtropical regions including Bangladesh. Currently, *R. communis* grows worldwide on industrial scale for the production of ricinoleic acid rich castor oil (seed contain 40 % oil) [15, 16]. Castor oil along with other castor products has wide range of industrial applications such as lubricants [16], cosmetics and plastics. Pharmacologically, *R. communis* has well been reported to possess strong anti-HIV [17], anticancer [18], contraceptive, purgative and laxative [19], anti-inflammatory [20], hepato-protective [21] and antioxidant [22] activity. Leaf essential oil and leaf methanol extract of castor plant have recently been reported to possess potent antibacterial, antifungal and leishmanicidal activity [23–25]. Isolated lectin, from the seed of *R. communis* has previously been reported to exert anti-proliferative activity against tumor cells both in vitro and in vivo condition [18, 26]. With the advancement of nanocarriers research, scientists are finding great prospect in cancer therapy with castor lectin (ricin) to selectively target the cancer cells using nanocarriers [27].

Seed proteins are small hydrophilic proteins, ranging from 83 to 153 amino acid residues, performing a number of crucial physiological functions mainly seed’s and sapling’s defense. Although the castor seed is the rich source of lectin (ricin), till date a limited study have been documented regarding to the antibacterial and in vivo anti-proliferative activities of crude protein of castor bean. Therefore, we designed this study to evaluate antibacterial and anti-proliferative activities of castor seed protein using mouse model containing EAC cells.

## Methods

### Collection of plant material

Mature branch along with ripen bean of six different cultivars of *R. communis* were collected from the surrounding area of Rajshahi, Bangladesh in 2013. The identity of the plant materials was verified by the taxonomist at the Botany department of the University of Rajshahi, Bangladesh. A voucher specimen (Accession number: 1332) was deposited at the National Herbarium Dhaka, Bangladesh. The six varieties are locally known as Cultivar Shabje (variety-1), Cultivar roktima (variety-2), Cultivar Dhushor (variety-3), Cultivar Lalchay (variety-4), Cultivar Badami (variety-5), Cultivar Shadate (variety- 6).

### Extraction of total protein

The methodology used for the extraction of total protein was described previously by Zeng and Dong [28]. In brief, the shade dried seed kernels (500 g) were blended with Tris-HCl buffer by mortar and spatula. Then the resultant materials were homogenized with Tris-HCl buffer (1 ml/10 mg) as well as β-mercapto ethanol (1 μl). The homogenized mixtures were then centrifuged at 10,000 rpm for 20 min and supernatant was stored at 4 °C.

### Spectrophotometric analysis

The crude protein solution (1 ml) was added to 5 ml of the alkaline solution and then mixed thoroughly for 10 min. An aliquot of 0.5 ml diluted Folin-Ciolteau’s reagent (Sigma, USA) was added rapidly in the mixture and kept for 30 min. The Optical density of extracted protein sample was measured by Folin-Lowary Method [29] at 650 nm. Bovine serum albumin (BSA) was used as standard. Those two varieties gave comparatively higher concentrations of *R. communis* seed protein were selected for future lectin, antimicrobial as well as anticancer study.

### SDS-PAGE analysis

The extracted crude protein from six castor varieties and a molecular weight marker were loaded carefully in individual gel lane and electrophoresis was carried out at constant voltage (300 volts, 30 mA) until the tracking dye reached the bottom. The gel was stained with 0.5 % coomassie brilliant (Sigma, USA) blue for 6 h with continuous shaking and washed with water. After completion of washing, the gel was kept in decolorizing solution until the background disappeared. The gel was then photographed by gel documentation system (Alphaimager mini, Taiwan).

### Determination of lectin activity

Hemmaglutination assay was carried out to determine the lectin activity of two castor varieties: variety-1 (V-1) and variety-3 (V-3) using a technique was previously described by Correia and Coelho [30]. Briefly, in a 96-well microtiter U plate, 50 μl protein samples was placed in the first well and then serially diluted into the successive wells with phosphate buffered saline (PBS), pH 7.4. Then, 50 μl of 2 % blood mice blood suspension was added in each wall. PBS alone was added with equal amount of blood as control. The microtiter plate was kept at 37 °C for 30 min and observed the agglutination of mice blood. Hemagglutinating activity was counted as agglutination of blood for the lowest concentration of extract.

### Antimicrobial activity

#### Microorganism and inoculums preparation

The bacterial cultures used in the study were three Gram negative bacteria including *Escherichia coli (*ATCC 25922)*, Pseudomonas aeruginosa* (ATCC 27853), and *Enterobacter aerogenes* (ATCC 29751) as well as one Gram positive bacteria: *Staphylococcus aureus* (ATCC 29213). All the test organisms were kindly provided by Microbiology Laboratory, Department of genetic engineering and biotechnology, University Rajshahi, Bangladesh. The test organisms were maintained on agar slant at 4 °C and subculture on a fresh agar plates. For disc diffusion and minimum inhibitory concentration (MIC) assay, bacterial liquid cultures were initiated by placing a loop of bacteria from the slant into 10 ml of LB media.

### Disk diffusion assays

Disc diffusion assay was conducted to detect the bacterial susceptibility to castor seed protein [31]. The test organisms (100 μl) were inoculated on the surface of solid agar medium (Muller Hinton agar). The crude proteins from V-1 and V-3 at the concentration of 50, 100, 200 and 400 μl/disc were impregnated on paper disc (6 μm). Then the agar plates containing microorganisms, soaked with paper discs were incubated at 37 ± 0.1 °C for 24 h. The inhibition of bacterial growth was evaluated by measuring the diameter (mm) of the clear zone around each disc (excluding the diameter of the wall). The disc pipette with streptomycin (10 μl/disc) was used as positive control. The protein concentrations that gave an inhibition zone of more than 10 mm were considered to be active and therefore their MIC was determined.

### Determination of minimum inhibitory concentration (MIC)

The MIC of the castor protein (V-1 and V-3) was determined by micro-dilution method in a sterile flat-bottom 96 well plates [32]. Standard (streptomycin, 1 mg/ml) along with stock solutions of crude protein were prepared in 100 % DMSO (Merck, Germany) at subsequent diluted concentration of 1000, 500, 250, 125, 62.5, 31.25, 15.62 and 7.8 μg/ml. Finally, 100 μl of diluted bacterial suspension was added to each well to achieve a concentration of 5 × 10^5^ cfu/ml. The plate was kept in an incubator at 37 °C for 24 h. Inhibition of bacterial growth was evaluated by addition 40 μl of 0.2 mg/ml p-iodonitroterazolium chloride (INT) indicator solution to every well and then incubated at 37 °C for 30 min. Microbial activity was indicated by the formation of a pink-red coloration while inhibition of growth was signaled by the persistence of a clear coloration. The color change was then assessed visually and observed the lowest concentration that prevented the color change was considered as the MIC.

### Determination of anticancer activity

#### Experimental animal and treatment

A total of thirty mature female Swiss albino mice (25–30 g) were purchased from the department of Pharmacy of Jahangernagar University, Dhaka, Bangladesh. The animals were kept in the animal house of the Department of Biochemistry and Molecular Biology, University of Rajshahi, Bangladesh.

EAC cells used in this study were kindly provided by Protein and Enzyme Laboratory, Department of Biochemistry and Molecular Biology, University of Rajshahi, Bangladesh. Mice injected with EAC cells on day zero were divided into two major groups: treated group and control group. For this therapeutic evaluation, 1.5x10^5^ EAC cells were inoculated into the peritoneal cavity of each mouse. Treated group was again divided into four subgroups (each group contains six animals): group 1, group 2, group 3 and group 4. Group 1 and 2 received the protein sample from V-1 at 50 and 100 μg/ml/d/mouse, respectively after 24 h of inoculation. Group 3 and 4 received the protein sample from V-3 at 50 and 100 μg/ml/d/mouse, respectively. In each case, the volume of the protein solutions (injected intraperitoneally) was 0.1 ml/d/mouse. Animals of the control group received 0.1 ml of 2 % DMSO/d/mouse. Treatment course was conducted for 6 days from day one.

### Determination of cell growth inhibition

The inhibition of EAC cells growth in vivo was conducted by the method previously described by Sur and Ganguly [33]. At the end of 6 days treatment, EAC cells were diluted intraperitoneally with normal saline (0.98 %) followed by harvesting with needle. Viable EAC cells were counted in a hemocytometer using trypan blue dye (Sigma, USA) with light microscope (optika, Italy). The cell growth inhibition was calculated using the following formula:

$$ \%\ \mathrm{Cell}\ \mathrm{growth}\ \mathrm{inhibition}\kern0.5em =\kern0.5em \left(1\hbox{-} \mathrm{T}\mathrm{w}/\mathrm{C}\mathrm{w}\right)\times 100 $$

Where,

Tw = Mean of number of EAC cells of the treated group of mice

Cw = mean of number of EAC cells of the control group of mice.

### Apoptosis assessment by DAPI staining

Harvested EAC cells (1 ml) from each mouse were centrifuged at 1200 rpm for 2 min and collect the plate from supernatant. The plate was then washed with PBS for each time followed by centrifugation at 1200 rpm for 2 min for three times. The resultant cells were incubated with 5 μl DAPI staining solution in the dark for 10 min with subsequent adding of PBS to the DAPI containing pellet and then centrifuged at 1200 rpm for 2 min. Finally, 200 μl PBS was added to the pellet and 10 μl of the supernatant was taken in a microscopic slide and observed the morphological changes of EAC cells with fluorescence microscope.

### Statistical analysis

Statistical analyses for the assessment of antimicrobial and anticancer activity of *R. communis* seed protein in comparison with control was performed using one way ANOVA and student’s *t*-test method. Data are expressed as mean ± SD (*n* = 3) for spectrophotometric and antibacterial study. Data are expressed as mean ± SD (*n* = 6) for anti-proliferative study. The significance was set at *P* < 0.05 and *P* < 0.01.

## Results

### Spectrophotometric determination of protein

The concentration of total protein in individual sample is shown in Table 1. The concentrations of seed protein of six varieties of *R. communis* were 0.31, 0.21, 0.35, 0.27, 0.24 and 0.21 mg/ml, respectively. Here, the concentration of *R. communis* seed protein was found higher in V-1 and V-3, comparing with the rest of varieties. Therefore, both of these varieties were selected for lectin, antimicrobial and anticancer screening study.

**Table 1:** Concentration of the protein samples isolated from six varieties of castor bean

Protein samples	Concentration (mg/ml)
V-1	0.31 ± 0.036
V-2	0.21 ± 0.026
V-3	0.35 ± 0.045
V-4	0.27 ± 0.020
V-5	0.24 ± 0.034
V-6	0.21 ± 0.026

Each value is represented as mean ± SD (*n* = 3)

### Determination of molecular weight by SDS-PAGE

**Fig. 1:**
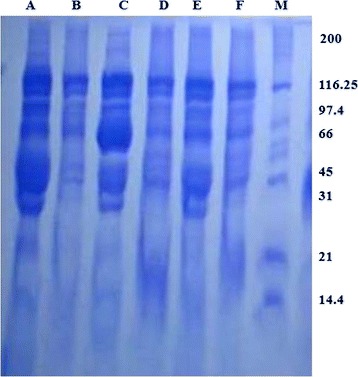
Gel electrophoresis of crude protein isolated from six varieties of castor bean grown in the Rajshahi region of Bangladesh: Lane-A: variety-1, B: variety-2, C: variety-3, D: variety-4, E: variety-5, F: variety-6 and M: molecular weight marker. Molecular weight of protein sample was determined as kDa

Molecular weight of total protein present in the castor bean was determined using the relative position of the standard molecular markers and the respective protein bands in the gel. In one dimensional SDS-PAGE, 14 bands with molecular weight ranging from 14.4–200 kDa were observed in all six varieties (Fig. 1). Among the all varieties a minor variations were observed in the banding patterns.

### Assessment of lectin activity

**Fig. 2:**
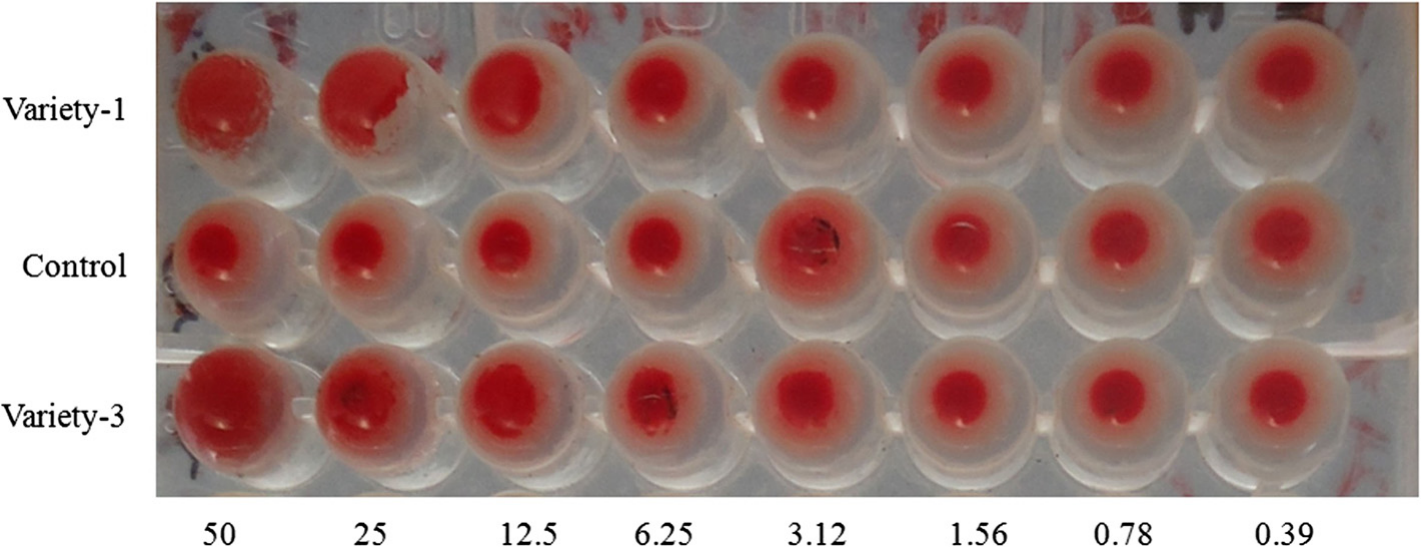
Hemagglutination activity of crud extract isolated from the bean of two castor verities (V-1 and V-3). Extract of V-1 hemagglutinated mice blood at 6.25 μg/wall, but in case of V-3 this activity was at 3.125 μg/wall. The control micro-titer-plate did not show hemagglutination at any concentration of PBS

The hemagglutination activity of both varieties castor protein (V-1 and V-3) is shown in Fig. 2. Both the varieties demonstrate hemagglutination activity on mice blood at different concentration. According to the data, the crude seed extract of V-3 shows comparatively higher agglutination activity (at 3.125 μg/wall) over extract of V-1 (at 6.24 μg/wall), indicating the presence of strong lectin activity in both varieties.

### Antimicrobial activity

#### Antibacterial activity of castor seed

Both of the castor varieties (V-1 and V-3) exhibits growth inhibitory activity against four pathogenic bacteria (Table 2). Data represent that, three bacterial species including *E coli, P aeruginosa* and *S. aureus* show significant susceptibility to castor bean protein even at low concentration (100 μl/disc), and the best result was observed for variety-3 against *S. aureus* (9.8 mm) at 50 μl/disc.

**Table 2:** Antibacterial activity of crude protein isolated from the seed of *R. communis* (V-1 and V-3) at different concentrations

Bacteria	Diameter of zone of inhibition (mm)								
	Variety-1				Variety-3				Antibiotic
	50	100	200	400	50	100	200	400	Strep
EC	--	8.9 ± 0.3	11.3 ± 0.4	17.7 ± 0.6	--	9.0 ± 0.8	12.8 ± 0.4	18 ± 0.7	22 ± 0.7
PA	--	9.3 ± 1.1	15.6 ± 0.6	18.8 ± 0.7	9.2 ± 0.6	10 ± 0.8	15.4 ± 0.5	19.5 ± 1.3	25 ± 0.4
EA	--	--	9.1 ± 0.9	11.8 ± 0.5	--	--	10.6 ± 0.6	12 ± 0.8	28 ± 1.4
SA	8.2 ± 0.8	10.4 ± 0.4	15.4 ± 0.6	21.4 ± 0.5	9.8 ± 0.4	12 ± 0.7	16.5 ± 1.6	23.1 ± 0.3	26 ± 0.8

Data represented as averages ± SD (*n* = 3); --, no measurable zone of inhibition; concentration of protein extract as μg/disc; EC: *E. coli*; PA: *P. aeruginosa*; EA: *E. aerogenes*; SA: *S. aureus* and Strep: Streptomycin

### Minimum inhibitory concentration (MIC)

The antibacterial activity of castor bean protein in terms of MIC is shown in Table 3. Among the four bacterial strains, the *R. communis* seed protein shows highest antibacterial activity against *S. aureus*, exhibiting the lowest MIC value (62.5 μg/ml for V-3), while *E. aerogenes* demonstrates comparative resistance to the crude of both varieties of *R. communis* and exhibits highest MIC value of 500 μg/ml for V-1. The MIC value for *E. coli* and *P. aeruginosa* were 250 and 125 μg/ml, respectively for both the castor varieties which indicates theses concentrations of *R. communis* seed protein would be able to inhibit the growth of these two bacterial species.

**Table 3:** Minimum inhibitory concentration of *R. communis* seed protein (V-1 and V-3) against the tested bacterial culture

Bacteria	Minimum Inhibitory Concentration (μg/ml)	
	Variety-1	Variety-3
*E. coli*	250	250
*P. aeruginosa*	125	125
*E. aerogenes*	500	250
*S. aureus*	125	62.5

Data represented as averages ± SD (*n* = 3)

### Anti-proliferative activity

#### Growth inhibitory activity of castor bean protein on EAC cells

**Fig. 3:**
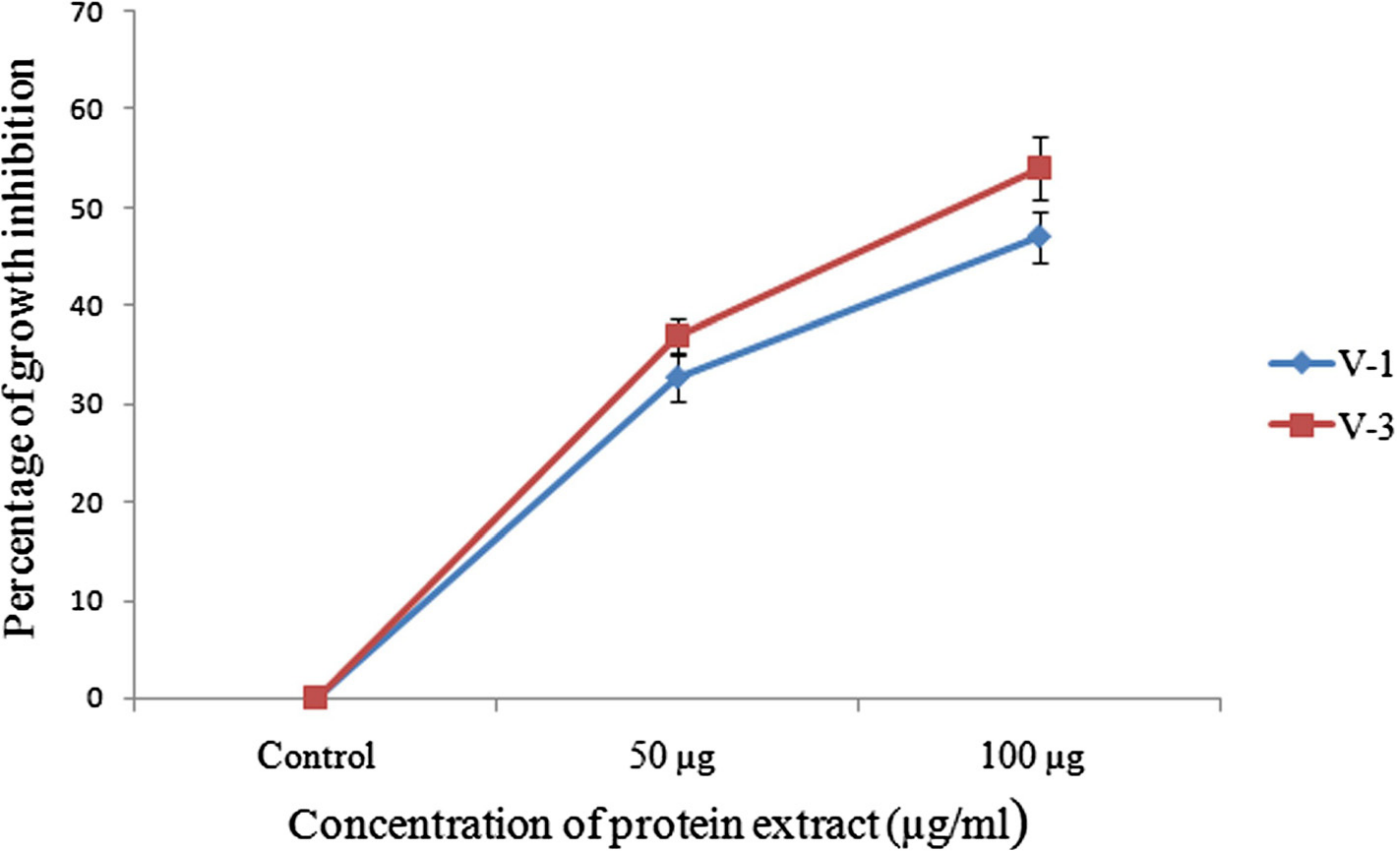
Growth inhibitory activity of *R. communis* seed protein on EAC cells. The graph shows the percentage of EAC cell death against two concentrations (50 and 100 μg/ml) of crude protein isolated from the seed of *R. communis* (counting the percentage in treated groups by considering zero cell death in control group). Each value represents as mean ± SD (*n* = 6)

The percentage of growth inhibition of EAC cell after ending of 6 days treatment is shown in Fig. 3. After the ending of treatment period, the viability of EAC cells was decreased considerably in the both treated groups in a concentration dependant manner and best result was observed for the protein sample of V-3 at 100 μg/ml. The V-3 demonstrates relatively higher growth inhibitory activity, exhibiting 37 and 54 % inhibition at 50 and 100 μg/ml, respectively over than V-1 (32 and 47 % at the same concentration of protein).

### Detection of apoptosic EAC cell by DAPI staining

**Fig. 4:**
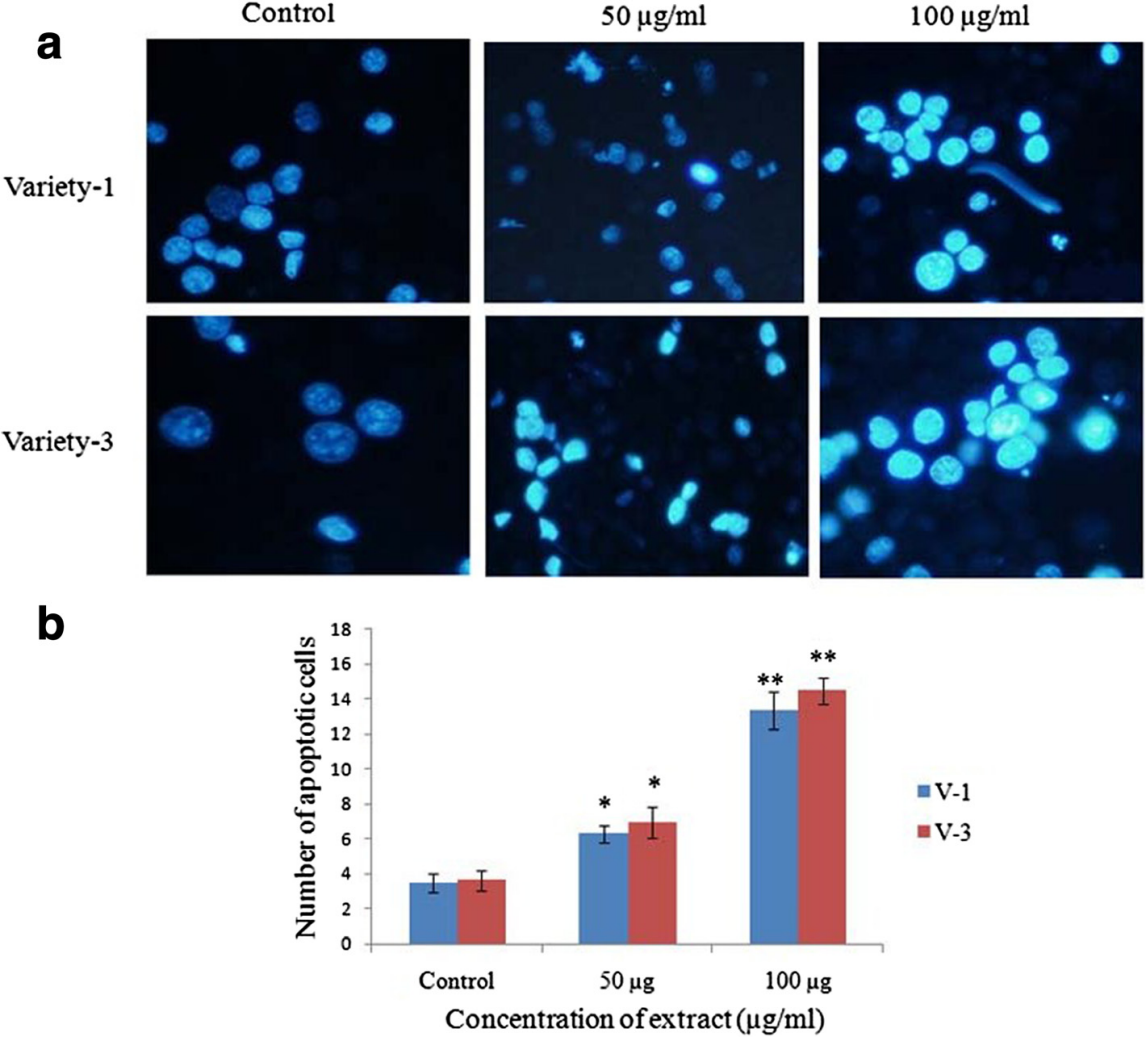
Detection of apoptotic cells through DAPI staining. **a** Normal cells are round in shape and show less bright blue fluorescence. Whereas the apoptotic cells are brightly stained with the characteristic features of apoptosis such as chromatin condensation, nuclear fragmentation and aggregation of apoptotic bodies etc. **b** Number of apoptotic cells per slide was estimated by counting apoptotic cells in five different fields. Each value represents a mean ± SD (*n* = 6). Significance was set at *P* < 0.05 (*) and *P* < 0.01 (**) with respect to control

The apoptotic alteration of EAC cells after 6 days of treatment is shown in Fig. 4a in comparison with regular and round shaped normal cells, stained with less bright blue fluorescence. Whereas the apoptotic cells or ongoing apoptotic cells exhibit bright blue color with condense chromatin. Reversely to the normal cells, the apoptotic cells exhibit characteristic apoptotic changes such as membrane blebbing, cell shrinkage, chromatin condensation, nuclear fragmentation and formation of apoptotic bodies (Fig. 4a). The average number of apoptotic cells/slide is shown in Fig. 4b. The average number of apoptotic cells in case of V-3 were 7 and 15 at 50 and 100 μg/ml, respectively, while this value were 6 and 13 for the same concentration of extract of V-1.

## Discussion

Plants are considered as the richest natural source to screen potential antimicrobial peptide (AMP) and phytochemicals. Besides the secondary metabolites, recently scientists have concentrated their attention to plant AMP which exhibits antagonistic action against wide range of microbes [34]. AMP can be considered as the potential agent to overcome drug resistance due to difference in the mode of their action from conventional antibiotics. Antibiotics generally penetrate into the cells without damaging cell wall and target to collapse specific cellular machineries such as inhibition of biosynthesis of peptidoglycan, inhibition of protein biosynthesis and breaking of double-stranded DNA etc [35]. Therefore, antibiotics are no longer able to interfere with such machineries due to the higher mutation rate of microorganisms, resulting drug resistance. In contrast, AMP in general penetrates into the bacteria by disrupting the cell wall and damage cell machineries regardless of specific targets [36]. Usually, AMP serves as nonspecific type of defense system, acting on a range of microbes through highly specific cell-mediated immune response based on the body requirement [37]. The normal immune responses of the body due to its slow activation and response are considered not enough to cope with the potential kinetics of microbial proliferation. Whereas, AMP can be activated promptly after the invasion of bacterial pathogen and neutralizes the pathogen rapidly.

In the present study, spectrophotometric analysis shows that the concentration of total crude protein extracted from the seed of six castor varieties range between 21–35 mg/ml (Table 1). The data reveal that the seed of V–1 and V-3 had higher concentration of protein then the rest of varieties. SDS-PAGE analysis also supports this finding by showing bolder protein band in V-1 and V-3 lane over then the other varieties, ranging from 14–200 kDa. The 66-kDa protein is lectin protein ricin (Fig. 1), comprising two distinct subunit active 32-kDa A chain (containing enzymatic activity) and 34-kDa B chain (lectin activity), joined by a disulfide bond [38].

The potent antimicrobial activity in the present study would be due to the action of lectin protein such as ricin in the castor bean. Lectin activity in plant sample is usually determined by hemagglutinating assay. Relatively higher antibacterial activity of V-3 is also supported by the moderately higher hemagglutination activity of of V-3 (3.12 μg/ml) over V-1 (6.25 μg/m). It has widely been reported that seed lectin possess potent growth inhibitory activity over a number of human pathogenic bacteria [39, 40]. Antimicrobial potentiality of plant lectin is mainly attributed to the preferential binding with carbohydrate molecules present on the bacterial cell wall. Lectins of different carbohydrate specificities can recognize a number of bacterial cell wall components mainly peptidoglycans (such as muramic acid, *N*-acetylmuramic acid, *N*acetylglucosamine etc) and lipopolysaccharides which strongly interact with lectin [40].

*R. communis* biomarker peptides-RCB-(1–3) has recently been synthesized artificially (RCB-1) and reported to possess considerable growth inhibitory activity against some pathogenic bacteria and fungi [41]. AMP usually targets broad range of bacteria due to the interaction of their reactive side chains with the surface molecules of bacterial cell wall. The outer surface of both Gram-negative (lipopolysaccharides) and Gram-positive bacteria (teichoic acids) have net negative charge, allowing them to preferential binding with the positively charged (preserved characteristic) AMP [42]. Upon binding, AMP creates pore on the bacterial membrane and collapse the transmembrane electrochemical gradients which lead to increase water and ion flow across the membrane, resulting cell swelling and osmolysis [36, 42]. The antibacterial activity of seed protein observed herein is considerably higher from the essential oil (except *S. aurous*), methanol and ethanol extract of castor leaf [23, 24, 43]. However, antimicrobial activity is the complex process. Therefore, it is likely that the antimicrobial activity observed herein would also be due to either the synergistic effect of the secondary metabolites alone (alkaloids, saponins, tannins, resins, flavonoids, terpenoids) present in extract or with AMP [43].

Apoptosis is an ideal way of cell death by which the body selectively eliminates unnecessary cells or unhealthy cells in a series of sequential events without affecting surrounding normal cells. Inhibition of apoptosis is the critical early event in tumor development, which allows the cell to proliferate abnormally and leading to the development of cancer. Therefore, induction of apoptosis is considered as the central strategy for almost every type of cancer treatment and prevention. The gradual falling of EAC cells number in treated mice would be the result of the induction of apoptosis which modulates cell numbers decline (Fig. 3) [44]. DAPI staining of EAC cells clearly demonstrates some morphological feature of apoptosis including membrane blebbing, cell shrinkage, chromosome condensation, nuclear fragmentation and aggregation of apoptotic bodies (Fig. 4).

However, potent lectin activity of the castor protein in this study would be the critical factor of strong anti-proliferative activity on EAC cells. Comparatively higher hemagglutination activity of V-3 also confirms the relatively higher anti-proliferative outcome (54 % growth inhibition) over the V-1 extract (47 %) in the current study (Fig. 3). Accumulation of evidences suggests that there is strong correlation between certain lectin-binding patterns and their biological behavior in various tumors [45]. Plant lectin in general exerts their apoptotic role by preferential binding to the cancer cell membrane which induces cytotoxicity [46]. Lectin also plays growth inhibitory role by altering the cell cycle and inducing G2/M phase cell cycle arrest and apoptosis [47].

It has previously been reported that *R. communis* lectin generates elevated level of reactive oxygen species (ROS) and induces DNA fragmentation [48, 49] which eventually up regulates p53 [50], the critical regulator of mitochondria mediated apoptosis. Upon over-expression, p53 induces up-regulation of pro-apoptotic protein “Bax” which in turn down regulates anti-apoptotic protein “Bcl-2”. The activated pro-apoptotic proteins creates pore on the outer surface of mitochondrial membrane followed by leaking the cytochrome *c* (Cyt *c*) into cytosol [51]. Mitochondrial Cyt *c* in cytosol binds with its binding partner apoptotic protease-activating factor- 1 (Apaf-1) which in turn binds with procaspase-9 and form a large wheel like multi-protein complex “apoptosome” [51]. The activated caspse-9 then cleaves the proenzyme forms of the effectors caspases “caspase-3”, the key executioner caspase of apoptotic mediated cell death which acts by restricted proteolysis of important cellular proteins [52]. It has widely been documented that plant lectin exerts their growth inhibitory role by inducing p53 mediated apoptosis [50]. Furthermore, one study shows that isolated lectin (ricin) from the seed of *R. communis* induces apoptosis in the cancer cells by up-regulating pro-apoptotic protein (Bak) and down-regulating Bcl-2 [53]. Therefore, it could be postulated that the crude protein isolated from castor been would produces elevated level of ROS which in turn modulates mitochondria mediated apoptosis of EAC cells.

Here, the antibacterial and growth inhibitory activity of *R. communis* seed protein could be attributed to the presence of a variety of bioactive protein especially lectin and secondary metabolites. Till date we do not know what bioactive polypeptide played the key antibacterial and anti-proliferative role on EAC cells. However, in future, we will identify, isolate and purify the protein of interest (lectin) responsible for current antibacterial and anticancer activity.

## Conclusion

Results from this study indicate that the concentration of crude protein isolated from six varieties of Bangladeshi castor bean ranged between 21–35 mg/ml and molecular weight between14–200 kDa. Both castor varieties (V-1 and V-3) show strong lectin, antibacterial as well as anti-proliferative activity. The concentration dependent decline in the number of EAC cells in treated mice observed here in was mainly due to the induction of apoptosis. However, the complete mechanism (in vitro study) underlying the therapeutic potential of the castor seed protein need to be investigated rigorously as an approach to develop fruitful combinational therapy.

## Abbreviation

AMP, Antimicrobial protein; DAPI, 4΄,6-diamidino-2-phenylindole; DMSO, Dimethyl sulfoxide; EAC, Ehrlich’s ascites carcinoma; kDa, Kilo Dalton; mg, milligramme; ml, milliliter; nm, nanometer; PBS, phosphate buffered saline; ROS, Reactive oxygen species; rpm, rotation per minutes; Tris-HCL, Tris- hydrochloric acid; SDS-PAGE, Sodium dodecyl sulphate polyacrylamide gel electrophoresis; μL, microliter; %, percentage and V, Variety
